# Influence of social mindfulness and Zhongyong thinking style on cooperative financial decision making in a Western sample

**DOI:** 10.1002/pchj.764

**Published:** 2024-05-22

**Authors:** Alexander Unger, Zixuan Li, Julie Papastamatelou, Chongzeng Bi

**Affiliations:** ^1^ East Asia Institute Ludwigshafen University of Business and Society Ludwigshafen Germany; ^2^ Mental Health Education Center Changsha University of Science and Technology Changsha China; ^3^ Study Program of Business Psychology University of Applied Management Studies (HdWM) Mannheim Germany; ^4^ Research Center for Psychology and Social Development, Faculty of Psychology Southwest University Chongqing China

**Keywords:** cooperative financial decision making, modesty, public goods game, social mindfulness, Zhongyong

## Abstract

Social mindfulness and Zhongyong thinking style are of high importance when evaluating relevant co‐actors in the social world. The current study investigates the influence of social mindfulness and Zhongyong thinking style on cooperative financial decision making in a public goods game among a Canadian sample. We hypothesize that higher perceived social mindfulness and higher perceived Zhongyong thinking style will increase the amount of money contributed to a joint project in a public goods game. The sample was a prolific‐based online recruited sample of *n* = 125 Canadians. We observed a significant main effect of Zhongyong thinking style on the amount of contributed money in the public goods game. Social mindfulness did not reach significance. The influence of Zhongyong thinking style was qualified by a significant Zhongyong by gender interaction, indicating that females but not males reduced their contributions if the Zhongyong thinking style of the co‐actor was manipulated as being low. It is shown that Zhongyong thinking style is also relevant in a Western cultural setting. Future research is needed, however, to investigate further the reasons for the differences between females and males.

## INTRODUCTION

The concept of Zhongyong thinking style refers to a specific Chinese mindset of re‐solving conflicts and complex problems (Zhou et al., 2021). A synonym of the concept is “doctrine of the mean,” which highlights one of the core ideas, namely to avoid extremes and seek for modesty in decision making and social relationships (Sin, 2023). Zhongyong thinking style has a long tradition in Chinese culture and is deeply rooted in Confucian philosophy (cf. Li, 2020). A related but less broad concept is social mindfulness, which is focused on interpersonal interactions. Individuals scoring high on social mindfulness take the interests and needs of other co‐actors into account (Van Doesum et al., 2018). While social mindfulness has been understood as a general and not as a culture‐bound psychological construct, it varies across different cultures (Van Doesum et al., 2021) and fosters prosocial tendencies (Kil et al., 2021).

In the current study, we were interested in testing the possibility of applying a Zhongyong experimental manipulation in a Western sample as a factor of influence on cooperative decision making in a public goods game (Kurzban & Houser, 2001). The intended combined operationalization with social mindfulness enables a critical testing of Zhongyong in a Western context. Considering that social mindfulness is a well‐established construct in the Western context, and depending on the results, we can draw important conclusions on the generalizability of the Zhongyong construct beyond a Chinese context.

### Zhongyong thinking style

An and Lee (2019) summarize the three core elements of Zhongyong, which are (a) timeliness and harmony, (b) dynamic balance and equilibrium, and (c) empathy. According to An and Lee (2019, p. 275), the first refers to “understanding the situations and taking appropriate action or optimal response […the second to] not being biased and going toward the extreme […and the third means] being open to and tolerant of others (p. 275).” Zhongyong thinking style has been studied from multiple perspectives. Several studies have investigated how Zhongyong thinking style affects entrepreneurial behavior (Li et al., 2019; Wei et al., 2020). Pan and Sun (2018) showed that Zhongyong thinking style affects employees' self‐regulation behavior, which becomes stronger if work tasks increase in complexity. Hou et al. (2020) demonstrated that Zhongyong thinking style is more pronounced among Chinese college students who experienced “less rejection and more warmth” (Hou et al., 2020, p. 1) from their parents. Those students with a higher pronounced Zhongyong thinking style showed, in turn, less distress. Yang et al. (2016) were able to show that Zhongyong also affects other domains of mental health. These authors reported that Chinese students scoring high on Zhongyong thinking had lower anxiety and depressive symptoms, and higher self‐esteem and life satisfaction compared with those scoring low on Zhongyong thinking. Similarly, Chou et al. (2014) reported the beneficial role of Zhongyong in coping with work stress and the well‐being of employees.

Beyond mental health and self‐regulation issues, the effects of Zhongyong have also been investigated for another aspect of human thinking and behaviors: Zhongyong thinking style has been shown to be important in personality development. For example, Lin et al. (2020) observed that Zhongyong thinking style mediates the enhancing influence of honesty–humility on the development of awe. Wei and Wang (2020) reported that Zhongyong thinking style enables, as a moderator, the transformation from crystallized intelligence to wisdom.

To summarize, Zhongyong thinking style is relevant in a wide range of research topics among Chinese samples. Further, the understanding of Zhongyong as a cognitive thinking style or mindset deeply rooted in Chinese culture makes the construct a very interesting issue, in particular for decision making (Li et al., 2019), problem solving (e.g., Zhou et al., 2021), and other cognitive processes such as remote‐association thinking (Zhou et al., 2019) or processing capacity (Chang & Yang, 2014).

Although Zhongyong can be seen as a genuine construct of Chinese culture, rooted deeply in Confucianism, similar wisdom and thinking can also be traced back to Greek philosophy, which must be seen as one of the primary roots of Western philosophy. For example, the virtue of modesty in Western thinking (Ben‐Ze'ew, 1993) is rooted in the concept of the golden mean (*chrysê mesótēs*) by Aristotle (Lawrenz, 2020; Losin, 1987). Related ideas are also expressed by Socrates in his advice to avoid the extremes and choose the mean, or in the famous tale of Daedalus and Icarus, in which Daedalus advised his son Icarus to “fly the middle course” and not to “fly too close to the sun” (Sadler‐Smith, 2020).

Other Western equivalents are found in folk wisdom, such as “it is better to bend than to break” or “the wiser head gives in”. Of course, the existence of these wisdom and thinking styles in antiquity does not mean that they are relevant nowadays. Their prevalence varies across societies, individuals, and situations.

Without ignoring the existence of substantial cultural differences between Chinese and Western cultures (e.g., Peng & Nisbett, 1999), we intend to apply the concept to a Western sample, because we assume that equivalent thinking styles might also exist among Western participants, which can be measured by the same items as in China. We do not assume that Zhongyong thinking style or very similar constructs are necessarily articulated the same way in Western cultures as in China. We assume, however, that many individuals might tend to modest positions and complex thinking in Western cultures. Support for our assumption of equivalent forms comes from a notable work by Li et al. (2019). Their theoretical framework addresses how Zhongyong can be highly relevant for strategic decisions in management in general.

Hitherto, research based on Western samples has been restricted to a few studies with related constructs such as critical thinking. Critical thinking style (Dahl et al., 2018; Zhang, 2003) can be seen as being equivalent to Zhongyong thinking style in Western cultures. Lun et al. (2010) conducted two experiments with Asian and Western students from New Zealand, showing that Western students can perform better in critical thinking. The authors explained these results by English proficiency rather than by the subtype of critical thinking, namely dialectical thinking style.

Based on the above‐outlined similarities in Chinese and Western philosophical thinking, we assume, in general, that forms equivalent to Zhongyong exist in Western countries. The current study intends to test if Zhongyong thinking style can affect decisions in a financial dilemma scenario.

### Social mindfulness

Van Doesum et al. (2013, p. 86) define social mindfulness as two‐dimensional “other‐regarding choices”, including skills and will. The first implies the ability of perspective taking, and the second implies being empathic and prosocial. These skills and the will “to act mindfully toward another person's control over outcomes” are the core concepts of social mindfulness (Van Doesum et al., 2013, p. 86). Contrary to their original assumption that social mindfulness requires specific situational prerequisites, such as the absence of time pressure or cognitive load, because it is assumed to be processed consciously, Mischkowski et al. (2018) observed that social mindfulness is entirely independent of experimental manipulations varying the process mode of the participants. Rather, Van Doesum et al. (2018) showed that the mere presence of one or several other persons is sufficient to activate greater social mindfulness. Another study by Lu et al. (2020) demonstrated that observing others behaving socially mindfully through relatively subtle gestures affected electroencephalogram (EEG) measurements, indicating that human detection of socially mindful behavior has a solid neuronal basis. This implies a high perceptual sensitivity in the perception of social mindfulness. Several authors emphasize that social mindfulness can be assumed to be more pronounced in collectivist cultures such as China or Japan (Markus & Kitayama, 1991a, 1991b; Zubair et al., 2018).

### The relationship between social mindfulness and Zhongyong thinking style

Social mindfulness is defined as a type of prosocial behavior in which the needs and wishes of other people are considered (Van Doesum et al., 2013; Van Lange & Van Doesum, 2015). In contrast to Zhongyong thinking style, it is not assumed to be a culture‐specific construct. Studies have been conducted in Asian as well as in Western cultures. According to a global study by Van Doesum et al. (2021) in 30 countries, there are substantial differences across different countries, but these differences at the country level did not result in higher scores in Asian countries than in Western countries. In fact, those countries with the highest social mindfulness score include Japan (72.0), but also European countries such as Austria (69.8) and the Czech Republic (68.8). Among those with the lowest score there are again Asian countries such as the Republic of Korea (56.4), as well as Western countries such as the United States (58.5).

When we consider the core element of the construct, which is being aware of the potential interests of other individuals and considering them in one's own behavior (e.g., choosing out of several objects in a way that the other person will not be restricted in his or her choosing options), it is evident that this kind of mindset is also part of the broader Zhongyong concept.

We intend to test the combined influence of the two constructs to investigate how the manipulations of both will influence cooperative behavior. This investigation is based on the importance of both constructs in research about cooperative behavior (cf. Lemmers‐Jansen et al., 2018; Ning et al., 2022). To the best of our knowledge, however, no studies have been published that combine the two constructs. Thus, the combination of the two manipulations fills an important gap by investigating the combined effects of Zhongyong and social mindfulness, which have both been observed to be important for cooperative financial behavior. If social mindfulness had effects but Zhongyong had no effects on cooperation in the public goods game, we should conclude that the Zhongyong manipulation depends on a Chinese cultural context. Instead, if all combinations of results show that Zhongyong increases cooperation in the public goods game, then we would allow the conclusion that Zhongyong is generalizable.

## PURPOSE OF THE STUDY AND HYPOTHESES

Mercier (2014) argues that Stoicism and Zhongyong have many similarities. As outlined above, there are several Western philosophies that have a strong similarity to Zhongyong, in particular in relation to ethical suggestions to be modest and seek compromises. Consequently, we assume that Zhongyong might be much more of a global construct than hitherto assumed. To summarize, our first argument for investigating Zhongyong in a Western context is that Western philosophy includes many equivalent approaches.

To further elaborate our argumentation, we refer to another aspect, which is associated with the measurement instrument applied in the current study. When we considered the measurement instrument, it turned out that the priming procedure consists of general elements and not of culture‐specific elements. The following two statements expressing a high Zhongyong orientation – “*When a problematic situation arises, one should remain calm, not make hasty decisions, but closely watch how it progresses*” and “*No matter how ‘right’ one thinks one is, being merciful to others is always a good virtue*” – can undoubtedly be applied in a Western country. Consequently, we argue that there are Zhongyong‐equivalent constructs in Western cultures.

To summarize, the aim of the current study is twofold. First, we intend to apply Zhongyong priming in a Western sample to test if Western participants are sensitive to it in a similar way to Chinese participants. Second, we intend to investigate if Zhongyong priming influences social cooperation in interaction with the priming of social mindfulness of an interaction partner. To our knowledge, the combination of the two constructs has never been tested in an experimental study.

Social mindfulness is characterizable as involving high thoughtfulness and social orientation. Both of these characteristics can be assumed to lead to a higher level of cooperation and a lower level of competitiveness and hostility. One central facet of Zhongyong thinking style is modesty and the consideration of the interests of others. Therefore, we assume that perceiving an interaction partner as socially mindful and showing a high level of Zhongyong thinking style characteristics will lead to higher cooperation (H1 and H2) and higher assumed cooperation of the interaction partner (H3 and H4). Trust in the interaction partner and a norm of mutually benevolent behavior can be assumed as linking elements. We also assume that cooperation and assumed cooperation will show a positive correlation. Cooperation will be operationalized as a cooperation scenario in which a certain amount of money can be contributed to a joint project. In summary, we will test the following hypotheses:

Perceived high socially mindful behavior of the interaction partner will increase the amount of money contributed to a joint project (reflecting more cooperation).

Perceived high Zhongyong thinking style of the interaction partner will increase the amount of money contributed to a joint project (reflecting more cooperation).

Perceived high socially mindful behavior of the interaction partner will increase the estimated amount of money contributed by the interaction partner (reflecting higher expected cooperation of the interaction partner).

Perceived high Zhongyong thinking style of the interaction partner will increase the estimated amount of money contributed by the interaction partner (reflecting higher expected cooperation of the interaction partner).

The two dependent measurements will be positively correlated.

In an exploratory way, we will test the existence of the interaction effects of perceived social mindfulness and Zhongyong thinking style. We will also include gender and status of studying (students vs. non‐students) as factors in the analyses of variance (ANOVAs) and test their effects and corresponding interaction effects in an exploratory way. We consider that gender and status of studying might influence how social mindfulness and Zhongyong thinking style constructs will be perceived without explicitly formulating hypotheses about their influence. There might be gender differences in the ability to detect social mindfulness and complex thinking‐related behavior. A similar potential difference might exist for students and non‐students and for individuals in different occupations.

## METHODS AND MATERIALS

We used an online‐based experimental design. For this purpose, we used the Profilic tool, which Palan and Schitter (2018) evaluated as a valuable, reliable online data‐collecting tool with a high emphasis on transparency. The experiment was programmed with *Qualtrics* (International Inc. Provo, UT).

### Participants

We conducted a g‐power analysis (Faul et al., 2009) to define an adequate sample size. The reported effect size by Dou et al. (2018) of η_p_^2^ = .20 for social mindfulness indicates quite a high effect size. We based our calculation on a somewhat more conservative assumption. We expanded our research design to include the influence of Zhongyong on financial decision making, about which no published effect sizes were available. For this reason, and because we tested our hypotheses with a Western sample (with a tendency to show somewhat lower effects), a lower assumed effect size seems reasonable. Another reason for the conservative assumption of a lower effect size of f = 0.35 (equivalent to η_p_^2^ = .11) is that assuming a smaller effect (with a correspondingly larger sample size) would only risk inadequate conclusions if the effect size was actually higher than assumed, which might lead to significant results even though the effect is insignificant (type II error). This can, however, be detected and considered if a higher effect size is observed.

We applied G‐Power version 3.1.9.4. (Faul et al., 2009). The adequate minimum sample size was calculated a priori based on the following assumptions: *f* = 0.35 (equivalent to η_p_^2^ = .11); Power (1‐prob = 0.95) = 0.95; *df* = 1, and number of groups =16 (with four 2‐level factors), resulting in a required sample size of *n* = 109 (Crit. *F* = 3.94; atual Power = .951). Because we surmised that a small number of participants would not understand the cooperating game, we increased the sample size to 161. Among the 161 recruited participants, 36 were precluded because they did not respond correctly to one or both control variables. The remaining sample size of *n* = 125 (50 female, 75 male) Canadian participants with a mean age of 29.4 years (*SD* = 9.4) constitutes a tolerable deviation from the intended sample size of *n* = 109. The sample consisted of 42 (33.6%) students enrolled in various courses of study, 67 (53.6%) employed participants, 15 (12%) unemployed participants, and 1 (0.8%) retired participant. Our only screening conditions were permanent residency in Canada and fluent English ability.

The sample sizes for the conditions for Zhongyong by gender were as follows: *n* = 23 females for low Zhongyong; *n* = 27 females for high Zhongyong; *n* = 38 males for low Zhongyong; and *n* = 37 for high Zhongyong. The chi‐squared test showed no significance (*p* = .371 for one‐tailed), indicating independence of the Zhongyong manipulation and gender. The corresponding sample sizes for each condition for social mindfulness by gender were as follows: *n* = 31 females for low social mindfulness; *n* = 19 females for high social mindfulness; *n* = 38 males for low social mindfulness; and *n* = 37 for high social mindfulness. Again, the chi‐squared test showed no significance (*p* = .143 for one‐tailed), indicating independence of the social mindfulness manipulation and gender.

When we considered the homogeneity of variances of the relevant factors Zhongyong, social mindfulness, and gender on the amount of contributed money in the public goods game, we detected no violations of the homogeneity of variances assumption. All Levene tests were insignificant: *p* = .339 (social mindfulness); *p* = .098 (Zhongyong); and *p* = .656 (gender).

### Procedure

The design of our experiment included the factors of social mindfulness (high vs. low) × Zhongyong thinking style (high vs. low). Further, we included gender (female vs. male) and status of studying (students vs. non‐students) as factors in the analysis, resulting in a 2 by 2 by 2 by 2 design. We assume differences in the importance and perception of social mindfulness and Zhongyang thinking style for both. All participants were randomly assigned to one of the resulting four experimental groups with a 2 by 2 factorial design of social mindfulness (high vs. low) by Zhongyong thinking style (high vs. low). Each participant was ostensibly interacting online with an anonymous counterpart. The other person was presented as behaving either socially mindfully or not.

Further, the other person was presented as scoring either high or low in the Zhongyong thinking style. Both manipulations were incorporated into a multi‐stage game described below in detail. The basic procedure was successfully applied to manipulate perceived low versus high mindfulness in interpersonal interactions by Dou et al. (2018). The first Part A of the current experiment, which is the manipulation of the perception of social mindfulness, is borrowed from a study by Dou et al. (2018). One difference from the Dou et al. (2018) study is that we did not operationalize an observer condition as described by Dou et al. (p. 99).

### Social mindfulness

As an introduction to and instruction for the first task, which served as a manipulation of the social mindfulness perception of the ostensible co‐actor, three screens were presented to the participants, on which it was explained that they had to interact with another randomly chosen participant in an object‐choosing game. Further, it was stated that it was randomly decided by the computer that the corresponding actual participant always got to choose second. In fact, all participants had to choose second, and either a socially mindful virtual participant or a non‐socially mindful virtual participant was displayed. Afterward, a starting site was delivered to the participants containing information on how to start task 1.

**FIGURE 1:**
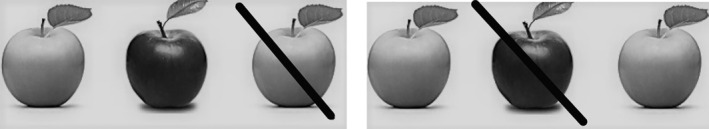
Example screen with (A) the social mindfulness condition on the left‐hand side and (B) the non‐social mindfulness condition on the right‐hand side (reproduced and modified from Lemmers‐Jansen et al., 2018).

The procedure of task 1 contains 12 rounds, in which in 8 out of 10 possible rounds, the co‐actor either chose one of the objects in a socially mindful way (leaving the actual choice to the participant) or in a non‐socially mindful way (leaving no actual choice to the participant, which means “choosing” between two identical objects; cf. Figure 1). Two rounds were created as being opposed to each condition. The remaining two rounds were filler rounds, in which the participants had the choice to choose between two types of objects independently from the decision of the co‐actor; this was done by presenting at least two of each object type. Both the opposition and the filler rounds were used to make the manipulation less evident to the participants.

Immediately after finishing task 1, 12 items (Dou et al., 2018) were presented with the following introduction: “Now you are supposed to answer some questions, press the key according to your feelings about the person one you just interacted with.” All 12 items were presented in a 7‐point Likert‐type response format ranging from 1 (= *Strongly Disagree*) to 7 (= *Strongly Agree*). Example items are “*Would you like to work with him/her*?” or “*It can be seen that he/she is selfish according to his behavior just now*.”

### Zhongyong thinking style

After task 1 and the following described manipulation check, task 2 (Part B) was introduced. Task 2 served as a manipulation of the perceived Zhongyong thinking style of the co‐actor as high or low. The responses of the participant to their own self‐evaluation of the Zhongyong thinking style items were used as filler tasks to mask the manipulation and indicate the Zhongyong thinking style of the co‐actor as either high or low.

All nine pairs of statements were taken from the Zhongyong Belief/Value Scale by Huang et al. (2012) (for a comprehensive review of the measurement instrument, see also Yang & Lin, 2014). Directly afterward, the participants filled out the manipulation checklist consisting of four items asking about how the co‐actor was perceived. These items measured to what extent the co‐actor was perceived as showing a high degree of Zhongyong thinking style. Again, the same 7‐point Likert‐type response format as for the other measurements was used. Next, the main dependent variable was the one‐shot public goods game (Part C). According to Fehr and Gächter (2000), public goods games are the standard procedure for measuring cooperation and social behaviors, and are “increasingly being used to measure social behaviors in humans and non‐human primates” (Burton‐Chellew et al., 2015, p. 1). Public goods games are also a well‐established measurement of financial cooperation (e.g. Dong et al., 2016; Houser & Kurzban, 2002) in repeated and one‐shot trials. In the current study, we applied the one‐shot version of a public goods game as described by Bilancini et al. (2022). Learning processes aimed at improving the corresponding individual payoff, as suggested by Burton‐Chellew et al. (2015) as an alternative explanation in repeated‐trial versions, can thus be widely ruled out in the current operationalization of a one‐shot trial. Further, Ackermann and Murphy (2019) emphasize that “The Slider Measure [which was applied in the current study] has been shown to be highly reliable and valid in terms of both its predictive power and its comparability to other […] measures” (p. 9).

In this public goods game, how much extra money would be donated to a joint project was measured. This was operationalized such that the participant only received extra money if the other ostensible participant cooperated too, and vice versa. Further, all money contributed to the joint project would be multiplied by 1.5 and then split evenly between the two players.

**FIGURE 2:**
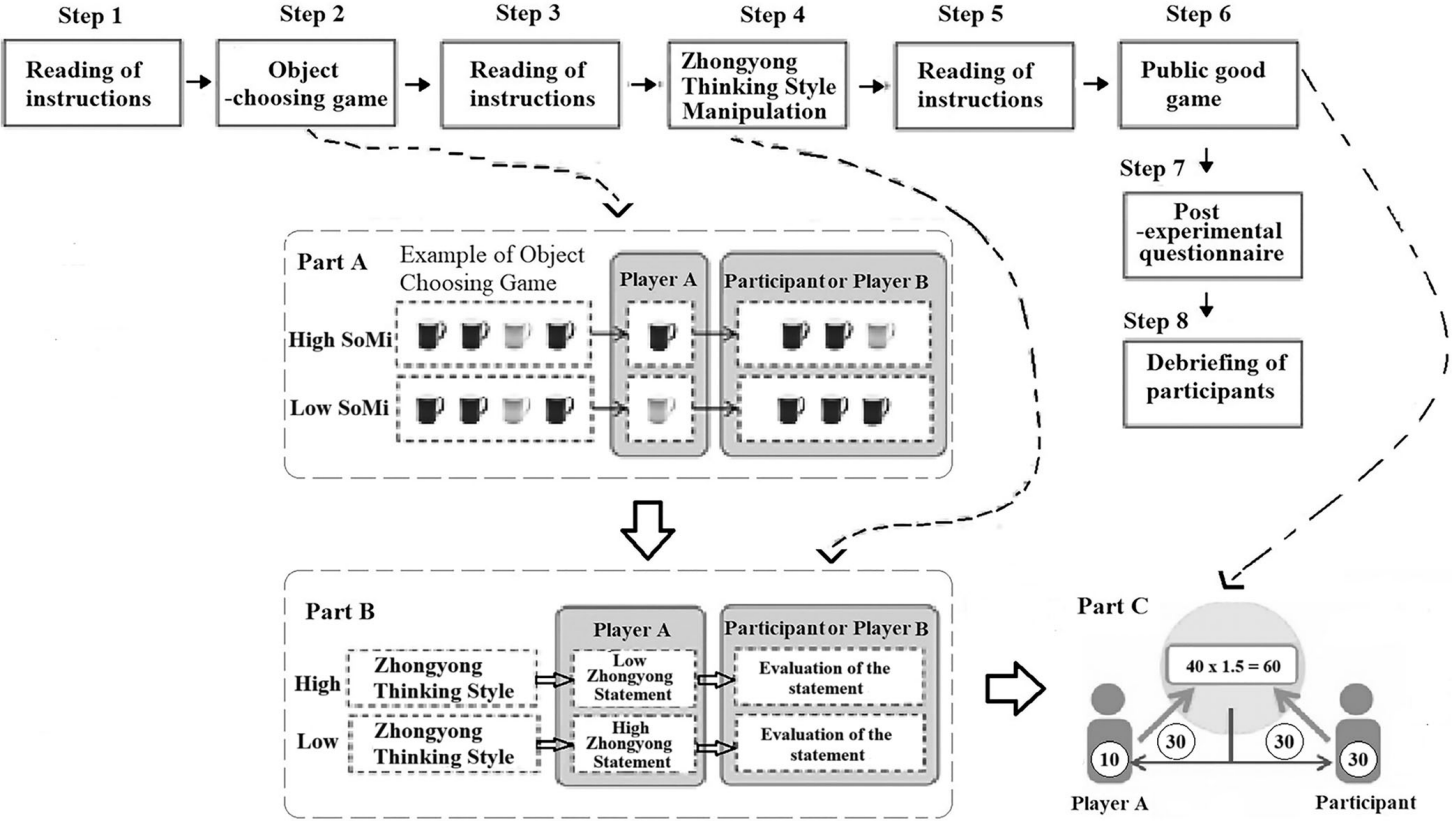
Schematic overview of the experimental procedure (heavily adapted and expanded from Dou et al., 2018, p. 100; fig. 1) with social mindfulness (SoMi) and Zhongyong thinking style.

Next, we asked about the estimated contribution of the co‐actor. The estimated contribution represents our second dependent measurement. Finally, we applied two control variables, which served to control for whether the instructions had been understood correctly. Participants who gave incorrect responses for both or one of the two control items were excluded from the analysis. A schematic overview of the procedure of the whole experiment is displayed in Figure 2.

## RESULTS

### Manipulation checks

All four items for the manipulation check for Zhongyong thinking style reached significance between conditions: holistic understanding (Item 1) (*M*_HIGH_ = 5.50, *SD* = 1.17 vs. *M*_LOW_ = 3.30, *SD* = 1.74, *p* < .001); seeing many sides and absence of excessive action (Item 2) (*M*_HIGH_ = 5.36, *SD* = 1.33 vs. *M*_LOW_ = 2.87, *SD* = 1.59, *p* < .001); handling things calmly (Item 3) (*M*_HIGH_ = 5.44, *SD* = 1.19 vs. *M*_Low_ = 3.36, *SD* = 1.50, *p* < .001); and compromising and giving when dealing (Item 4) (*M*_HIGH_ = 4.77, *SD* = 1.39 vs. *M*_Low_ = 2.41, *SD* = 1.49, *p* < .001). Thus, the Zhongyong thinking style perception of the interaction partner as high versus low was successfully induced.

Overall, we used 12 items as a manipulation check for social mindfulness (Dou et al., 2018). This manipulation check consisted of four sub‐scales, measuring *Willingness to collaborate*, *Desire to meet in real life*, *Liking*, and *Perceived self‐interestedness*. Each of these sub‐scales consisted of three items, and for each one a mean index was calculated, indicating a high perceived social mindfulness and a low perceived social mindfulness. The social mindfulness (SoMi) manipulation was observed to be significant in accordance with *Willingness to collaborate* (*M*_LOW SoMi_ = 3.97, *SD* = 1.27 vs. *M*_HIGH SoMi_ = 4.71, *SD* = 1.00, *p* < .01), *Liking* (*M*_LOW SoMi_ = 4.78, *SD* = 1.07 vs. *M*_HIGH SoMi_ = 5.34, *SD* = 0.81, *p* < .01) and *Perceived self‐interestedness* (*M*_LOW SoMi_ = 4.36, *SD* = 1.19 vs. *M*_HIGH SoMi_ = 3.39, *SD* = 0.92, *p* < .01). Only the *Desire to meet in real life* did not reach significance, *M*_LOW SoMi_ = 3.15 (*SD* = 1.41) vs. *M*_HIGH SoMi_ = 3.34 (*SD* = 1.09), *p* = .41. Overall, these results indicate that the social mindfulness manipulation can be assumed to be successful. The aspect of the desire to meet in real life might be inappropriate for measuring social mindfulness, at least for the current sample, or the non‐significant results might reflect some (minor) weakness of our manipulation.

### Main analysis

We conducted two ANOVAs including social mindfulness, Zhongyong thinking style, gender, and status of studying as factors predicting the amount of contributed money (to test H1 and H2) and predicting the estimation of how much the co‐actor would contribute (to test H3 and H4).

**FIGURE 3:**
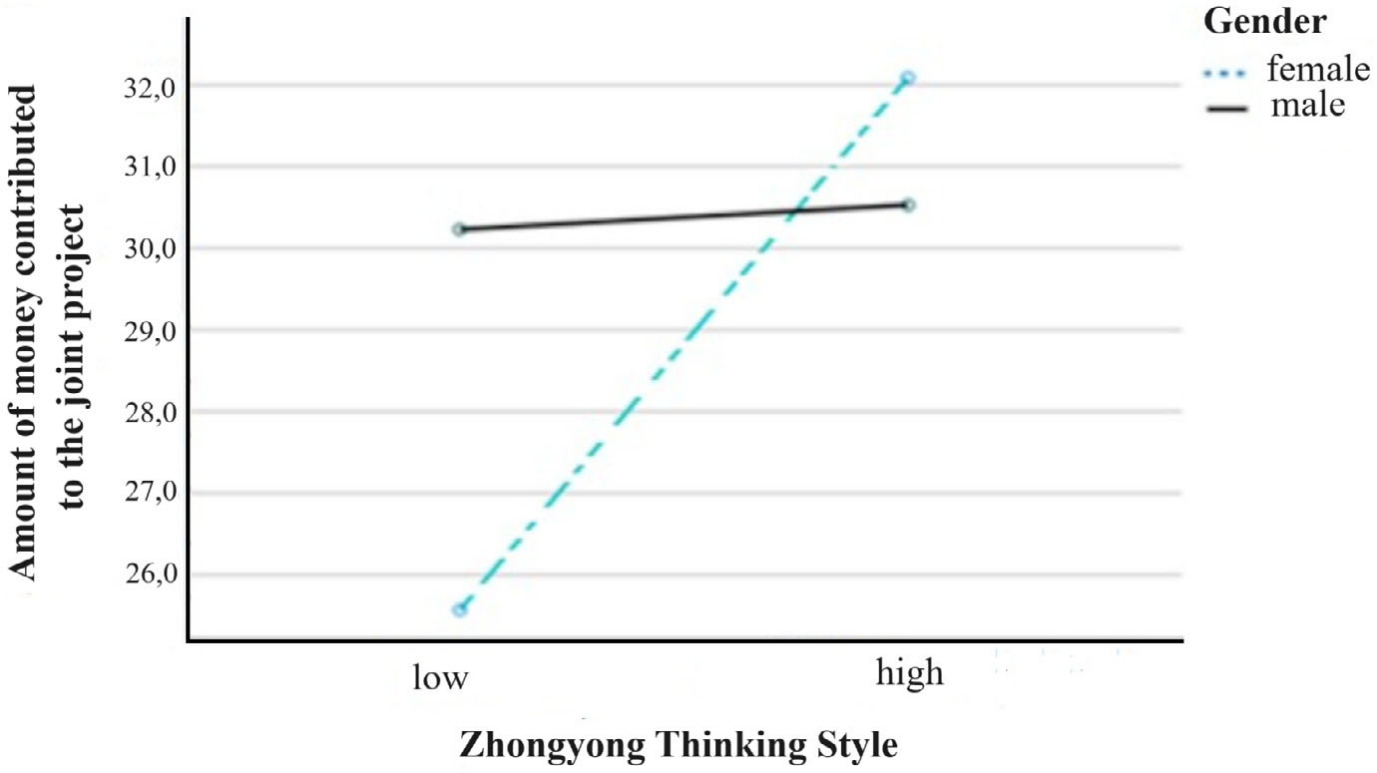
Zhongyong thinking style by gender on the contributed amount of money in the public goods game.

The first ANOVA on the amount of contributed money for the common project revealed a significant main effect of Zhongyong thinking style in the predicted direction [*F*(1, 104) = 5.36, *p* = .02, η^2^ = .05] and a significant two‐way interaction of Zhongyong thinking style by gender [*F*(2, 103) = 4.46, *p* = .04, η^2^ = .04]. All other main effects or interactions did not reach significance (all *p*s > .08). Thus, H2 was confirmed but not H1. For means and standard deviations, see Table 1. The observed interactions will be examined in more detail by planned contrast analysis analyzing the observed two‐way interaction of Zhongyong thinking style by gender (cf. also Figure 3).

**TABLE 1:** Means and standard deviations for contributed money by social mindfulness (SoMi), Zhongyong thinking style (ZTS), and the interaction gender × Zhongyong thinking style (ZTS).

Experimental condition	Low	High
Social mindfulness (SoMi)	*M* = 29.30 (14.03)	*M* = 31.20 (13.30)
Zhongyong thinking style (ZTS)	*M* = 28.61 (14.82)	*M* = 31.66 (12.42)

Zhongyong thinking style (ZTS)	Low	High
Females	*M* = 25.59 (15.63)	*M* = 32.58 (11.89)
Males	*M* = 30.40 (14.22)	*M* = 30.97 (12.93)

*Note*: Only significant interactions are displayed; standard deviations (*SD*) are displayed in parentheses.

We observed that the planned contrasts of level 1 (female and low Zhongyong) versus levels 2, 3, and 4 reached significance (*p* = .04). All other planned contrasts were non‐significant (all *p*s > .06). Thus, we can summarize that females in the low Zhongyong thinking style condition are significantly contributing less money compared with the other three groups. In contrast, those three groups showed no significant differences.

For the second ANOVA, on the amount of money estimated to be contributed by the interaction partner for the joint project, we observed significant main effects in the predicted directions for social mindfulness [*F*(1, 104) = 4.44; *p* = .04, η^2^ = .04] and Zhongyong thinking style [*F*(1, 104) = 25.34, *p* < .01, η^2^ = .20], confirming H3 and H4. We also observed a significant main effect of gender, *F*(1, 104) = 4.74, *p* = .03, η^2^ = .04, with higher estimations by males. Further, we observed a significant two‐way interaction of Zhongyong thinking style by gender [*F*(2, 103) = 6.89, *p* = .01, η^2^ = .06] and a three‐way interaction of social mindfulness by Zhongyong thinking style by gender [*F*(3, 102) = 5.37, *p* = .02, η^2^ = .05] (for descriptive statistics see Table 2).

**TABLE 2:** Means and standard deviations for estimated contributed money by social mindfulness (SoMi), Zhongyong thinking style (ZTS), and the interaction gender × Zhongyong thinking style (ZTS).

Experimental condition	Low	High
Social mindfulness (SoMi)	*M* = 23.85 (14.59)	*M* = 29.43 (13.62)
Zhongyong thinking style (ZTS)	*M* = 21.14 (15.89)	*M* = 31.00 (11.05)

Gender	Females	Males
	*M* = 23.52 (15.39)	*M* = 28.05 (13.54)

Zhongyong thinking style (ZTS)	Low	High
Females	*M* = 14.45 (14.45)	*M* = 30.50 (12.29)
Males	*M* = 24.76 (15.62)	*M* = 31.35 (10.26)

Social mindfulness (SoMi)	Low		High	
Zhongyong thinking style (ZTS)	Low	High	Low	High
Females	*M* = 14.08 (13.36)	*M* = 26.23 (13.21)	*M* = 15.00 (16.90)	*M* = 38.55 (3.36)
Males	*M* = 18.50 (16.70)	*M* = 32.50 (8.27)	*M* = 30.68 (12.19)	*M* = 30.00 (12.33)

*Note*: Only significant interactions are displayed; standard deviations (*SD*) are displayed in parentheses.

**FIGURE 4:**
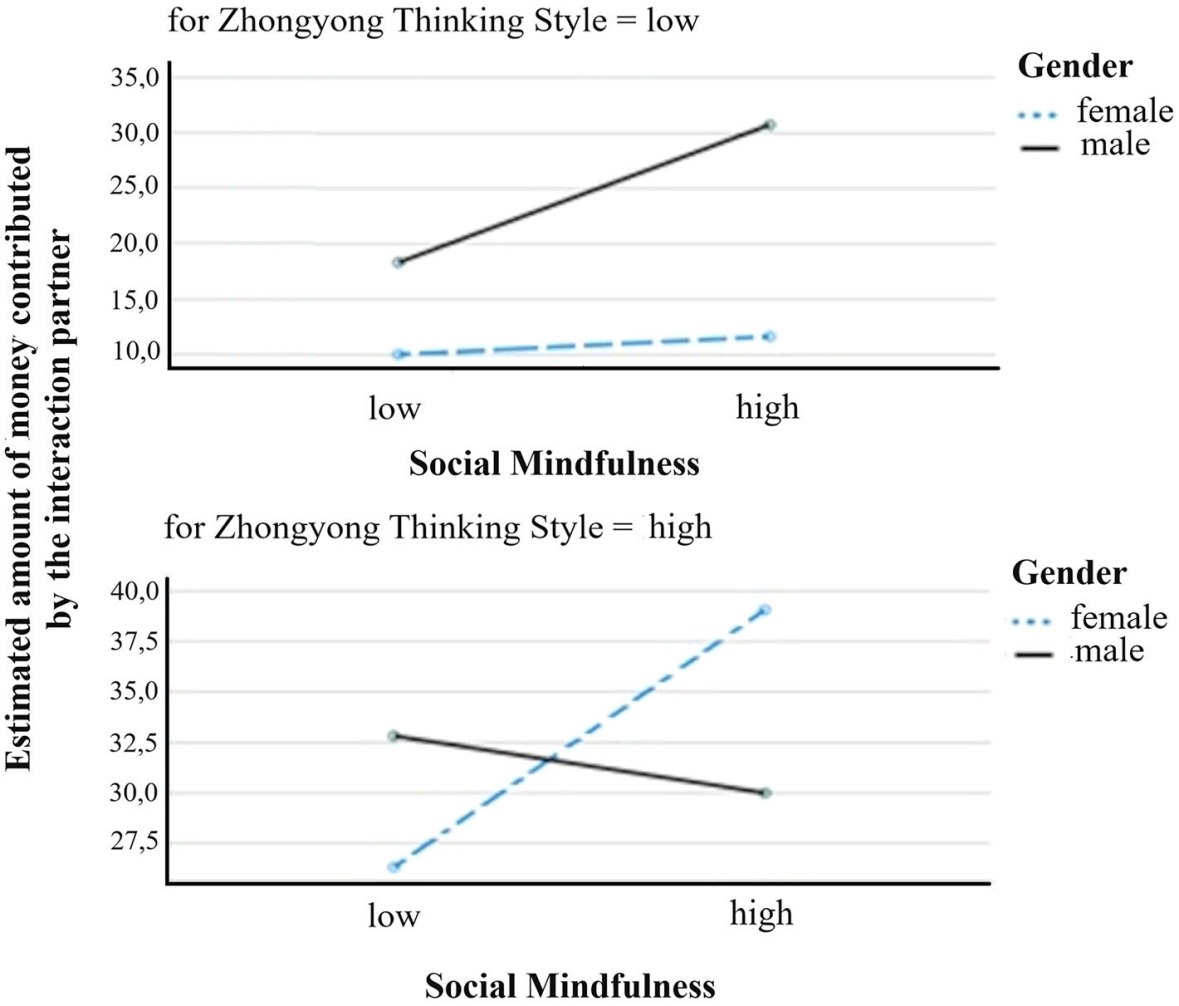
Social mindfulness by gender on the estimated amount of money contributed by the interaction partner for low and high Zhongyong thinking style.

The hypotheses about the increasing effect of social mindfulness (H3) and Zhongyong thinking style (H4) were confirmed. We further analyzed the observed three‐way interaction (presented in Figure 4) by calculating separately planned contrasts for high versus low Zhongyong thinking style with the following level coding: 1 = female and low social mindfulness; 2 = female and high social mindfulness; 3 = male and low social mindfulness; and 4 = male and high social mindfulness.

For the condition of low Zhongyong thinking style, the planned contrast analysis between level 2 (the females with high social mindfulness) to be lower than level 4 (the males with high social mindfulness) reached significance (*p* < .01; one‐tailed). All other planned contrasts analysis were non‐significant (all *p*s > .07; one‐tailed).

For the condition Zhongyong thinking style = high, we observed 3 out of 4 significant planned contrasts (all one‐tailed), which are:

1. the planned contrast of level 1 (females with low social mindfulness) to be lower compared to all other levels 2, 3, and 4 (*p* = .02);
2. the planned contrast of level 1 (females with low social mindfulness) to be significantly (*p* < .01) lower than level 2 (females with high social mindfulness) and
3. the planned contrast of level 2 (females with high social mindfulness) to be significantly lower than level 4 (males with high social mindfulness).

The planned contrast analysis between level 1 (females with low social mindfulness) to be lower than level 2 (females with high social mindfulness) showed only a non‐significant trend (*p* = .05).

According to the hypothesized positive correlation of both dependent measurements (H5), we observed, as predicted, a positive correlation between the contributed amount and the estimated amount of the interaction partner (*r* = .55, *p* < .01).

## DISCUSSION

We summarize the main results of our study as follows. First, we observed the main effects on the amount of money contributed to the Zhongyong thinking style. Those in the high Zhongyong thinking style condition contributed, as predicted, more money to the joint project compared with those in the low Zhongyong thinking style condition. Contrary to what was predicted, social mindfulness showed no main effect. The observed main effect of the Zhongyong thinking style was qualified by a Zhongyong thinking style by gender interaction. All observed interactions by gender must be interpreted cautiously, and all conclusions have a preliminary character. This is the case for both dependent measurements.

Accordingly, women were more affected by the Zhongyong thinking style manipulation, whereas men showed no significant difference between the conditions of high versus low Zhongyong thinking style. Both male groups contributed a relatively high amount of money. This presumably reflects ignorance of the information provided about the interaction partner or the estimation that the complex thinking style as presented is not relevant to behavior in the decision scenario by the male participants. A higher level of risk‐taking of the male participants is another possibility to explain the observed gender differences. Both conclusions are speculative and need to be tested in future research.

A similar but not identical pattern of influence was observed for the estimated amount of money contributed by the other interacting player. We observed the main effects of Zhongyong thinking style and social mindfulness in the predicted directions, but also a main effect of gender: the condition of high Zhongyong thinking style again enhanced the estimated amount. The condition of high social mindfulness showed the same enhancing effect, and women estimated the amount as much smaller compared with men. Most importantly, these main effects were qualified by a two‐way and three‐way interaction.

Against the background of the reported planned contrasts and the complex three‐way interaction (see Figure 2), we give the following preliminary possible interpretation: males' estimates are in no way affected by the factor of social mindfulness. The estimates vary only by small margins for low and high social mindfulness within each of the conditions of high versus low Zhongyong thinking style. Additional analyses revealed, however, that social mindfulness had an interaction effect with Zhongyong thinking style within the group of males, indicating that males recognized social mindfulness for their estimate. For males, there is a main effect of Zhongyong thinking style, with higher estimates in the high Zhongyong thinking style condition. Overall, male estimates were higher than female estimates except for the combined condition of high social mindfulness high Zhongyong thinking style. Females instead show lower estimates in three out of four conditions compared with men. They only had higher estimates than men if both social mindfulness and Zhongyong thinking style were high. Further, if Zhongyong thinking style was low, high social mindfulness had no enhancing effects on the female estimates. Social mindfulness was essential only if Zhongyong thinking style was high. As stated, all interpretations involving gender in interactions should be evaluated as strictly preliminary and can only be seen as a starting point for a more systematic testing with more gender‐balanced samples. The gender imbalance in the current study limits the interpretability of our results.

The current study has shown that Zhongyong thinking style, rather than social mindfulness, influenced the amount of money contributed in a cooperation scenario in a Western sample. For the estimated amount contributed by the interaction partner, both factors of influence (Zhongyong thinking style and social mindfulness) were shown to be relevant. The influence on both dependent variables was, as described above, quite different for females and males.

The explanations offered for the observed differences in male and female participants need to be analyzed further in future studies. The differences could also be explained by a lower compliance of the male participants with the instructions or by a lower level of information processing by the males.

The study shows that the manipulation of Zhongyong thinking style leads to the predicted change in cooperation behavior among a Western sample. However, the Zhongyong thinking style was operationalized explicitly as a culture‐specific Chinese construct in the current experiment. Thus, another important conclusion from this observation is that, in general, complex thinking seems to be a global construct that has equivalents in different cultures. In particular, the observation that social mindfulness did not increase the amount of contributed money in the public goods game while Zhongyong did is evidence for the assumed existence of Zhongyong equivalents in Western cultures.

However, several limitations have to be considered. One of the limitations of the current study is that we did not compare Western with Chinese participants. Such an approach could help detect essential differences and resolve the question about the limitation of generalizing complex thinking styles. Further, in the context of cross‐cultural futures studies, it would be interesting to analyze whether Zhongyong thinking style (or its Western equivalents) shows an overlap with social mindfulness beyond theoretical considerations and definitions. Another limitation of the current study that should be addressed in future studies relates to in which ways the participants vary in their Zhongyong thinking style proneness and social mindfulness (at the trait or situational level). In contrast to Dou et al. (2018), we did not observe the predicted influence of social mindfulness on the amount of contributed money. Two possible explanations seem reasonable. First, social mindfulness did not influence our Western sample because of its lower importance in Western cultures compared with in Asian cultures. This aligns with previous wide‐ranging country‐comparing studies (Kirkland et al., 2022; Van Doesum et al., 2021). A second explanation, however, is that the combined experimental design prevents a successful social mindfulness manipulation. It is possible that the following Zhongyong manipulation disguised the preceding social mindfulness manipulation. Participants might show a prevailing attentional shift toward the aspects of Zhongyong thinking style. Thus, it is a limitation of our current study that we did not vary the sequence of manipulations or add conditions with either Zhongyong or social mindfulness manipulations only. We recommend expanding the systematic testing of both constructs as described in future studies.

## CONCLUSIONS

Notwithstanding the above‐mentioned limitations, the current study allows the following two new and important main conclusions.

First, the current study showed that even deep, culturally rooted constructs such as Zhongyong thinking style can affect the cooperation behavior of Western individuals. Second, social mindfulness was of minor importance for cooperative behavior for a Western sample, although this needs to be re‐tested more systematically. We encourage follow‐up and future studies as outlined above to further investigate the discussed open questions and the observed specific results, such as the difference between genders in the perception of social mindfulness in a Western sample. Cross‐cultural studies, including Asian and non‐Asian samples, could also broaden the understanding of social mindfulness and the role of Zhongyong thinking style in cooperative financial decision making.

## CONFLICT OF INTEREST STATEMENT

The authors declare that there are no conflicts of interest.

## ETHICS STATEMENT

The study was approved by the Ethics Committee of the Faculty of Psychology, Southwest University. Informed consent was obtained from all participants included in the study.

## Supporting information


**Appendix S1.** Experimental material of part A.

**Appendix S2.** Experimental material of part B.

**Appendix S3.** Dependent Measurements in part C.

**Appendix S4.** ANOVAs on contributed amount of money for the common project and on the estimated level of money Amount contributed by the co‐actor for the common project.
