# Supplementary material for: Influence of social mindfulness and Zhongyong thinking style on cooperative financial decision making in a Western sample
Source: Psych J. 2024 May 22;13(5):749–59. doi: 10.1002/pchj.764 (PMC11444720; doi:10.1002/pchj.764)
Supplement: Supplementary file 1 — Appendix S1. Experimental material of part A. [file PCHJ-13-749-s001.docx]

**Appendix A : Experimental material of Part A**

1. **Manipulation of Social Mindfulness**

On **Screen 1** the following text was displayed:

*“Thank you for accepting this assignment. You have received C$.5 for participating. You also have the opportunity to receive additional money, which will be described in the next few pages.”*

**Screen 2** contains the following text:

*“You have been randomly assigned to interact with a stranger. All the task you are about to perform involves you two people. Both of you receive this same set of instructions. You cannot participate in this study more than once.”*

**Screen 3** contains the instruction for task 1:

*“Task1: You will play a few rounds in which we will show you some objects. If both of you were allowed to take one of these objects, which one would you choose? The computer has decided that you always get to choose* ***secondly*** *and that the objects he/she chooses will no longer be available to you."*

**Screen 4** contains an example screen about the choice situation after the choice of the co-actor with the following description:

*“First, here’s an example. Which one of these objects would you choose (type the number below)? Remember, the object with lines means it has been chosen by the other, and you can't choose."*

**Screen 5** contained information on how to start task 1:

*"Now, we will start the task*

*Remember that there are two of you, and that you get to choose* ***second****. (You may need to wait for a while.)*

*The choices you make now will affect the next steps.”*

On **Screen 6**, the stimulus material of the Social Mindfulness manipulation was displayed successively (cf. Dou et al., 2018).

**Screen 7** contains the following text:

*Now you are supposed to answer some questions, press the key according to your feelings about* ***the one*** *you just interacted with.*

This was followed by the manipulation check of Social Mindfulness (12 items by Dou et al., 2018) with a response format ranging from 1 = strongly disagree to 7 = strongly agree.
