# Supplementary material for: Influence of social mindfulness and Zhongyong thinking style on cooperative financial decision making in a Western sample
Source: Psych J. 2024 May 22;13(5):749–59. doi: 10.1002/pchj.764 (PMC11444720; doi:10.1002/pchj.764)
Supplement: Supplementary file 2 — Appendix S2. Experimental material of part B. [file PCHJ-13-749-s002.docx]

**Appendix B: Experimental material of Part B**

1. **The instruction for the manipulation of Zhongyong Thinking Style**

**Screen 8** contains the following instruction:

“*In this task, you are supposed to know more about the one you are interacting with. Here are nine pairs of sentences with* ***opposite*** *meanings; he/she will choose one of the sentences with which he/she agrees more; you should read this sentence carefully and* ***respond to the statement that he/she chose*** *by indicating how much you agree or disagree with it using the following rating scale. (You may need to wait for a while).”*

1. **The description of the Zhongyong Manipulation (not displayed to the participants)**

In each of the nine couples of statements (on **Screen 9**), one presents a high Zhongyong thinking style, whereas the other presents a low Zhongyong thinking style. The ostensible co-actor chooses either choose all high or all low Zhongyong thinking style statements. Each couple of statements appeared on the screen for 5 seconds, so the participants had enough time to read. After 5 seconds, the non-chosen disappeared, and the participants could indicate how much they agreed to the chosen statement in a 7-point Likert response format ranging from 1 (= *Strongly Disagree*) to 7 (= *Strongly Agree*). It was consistently displayed to the participants as the "response” of the co-actor, either the low or the high version of each of the nine pairs of items. For the The Manipulation of Zhongyong (pairs of nine items) pairs of nine items cf. Huang et al. (2012). **Screen 10** contains 4 items by Huang et al. (2012) as manipulation check with a response format ranging from 1 = strongly disagree to 7 = strongly agree.
