# Supplementary material for: Influence of social mindfulness and Zhongyong thinking style on cooperative financial decision making in a Western sample
Source: Psych J. 2024 May 22;13(5):749–59. doi: 10.1002/pchj.764 (PMC11444720; doi:10.1002/pchj.764)
Supplement: Supplementary file 3 — Appendix S3. Dependent Measurements in part C. [file PCHJ-13-749-s004.docx]

**Appendix C: Dependent Measurements in Part C**

**1. Contributed money to the joint project**

The exact formulation on **Screen 11** was as follows:

“*This is the last task, you two will be given 40 cents for this interaction (in addition to the basic payment you received already for participating). You each decide how much of your 40 cents to keep for yourself and how much (if any) to contribute to the group's common project.* ***All money contributed to the common project will be multiplied by 1.5 times and then split evenly between you two****. Thus, if both of you contribute all of your 40 cents, each of you will earn 60 cents; But if he/she contributes his/her 40 cents while you keep your 40 cents, you will earn 70 cents, while he/she only gets 30 cents; meanwhile, if you contribute all your 40 cents while he/she keeps his/her 40 cents, he/she will earn 70 cents while you only get 30 cents. Once you two have chosen how much to contribute, the interaction is over.*”

Further, the response format for the decision of how much of the 40 cents will be contributed was displayed **on Screen 12** as follows:

Please use the slider to choose the amount of money you wish to contribute.

Your contribution: 0----------------slider----------------40

1. **Estimation of the contributed money of the co-actor:**

The exact formulation on **Screen 13** was as follows:

“*In this stage, we would like you to predict the contribution of the one you just interacted with. You can earn up to an additional 40 cents depending on the accuracy of your prediction. Thus, you are incentivized to be as accurate as possible when making your prediction".*

And again, the slider format was used as displayed as follows (on **Screen 14**):

How much do you think the other contributed (0-40 cents)?

His/her contribution: 0----------------slider----------------40

1. **Control variables:**

These two control variables from Rand et al. (2015) were introduced on **Screen 15** with the following sentence: “*You must answer these two questions correctly to receive your bonus!*”.

The wording of both control items was:

“*What level of contribution earns the highest payoff for the group as a whole?”* and “*What level of contribution earns the highest payoff for you personally?*
