# Supplementary material for: Influence of social mindfulness and Zhongyong thinking style on cooperative financial decision making in a Western sample
Source: Psych J. 2024 May 22;13(5):749–59. doi: 10.1002/pchj.764 (PMC11444720; doi:10.1002/pchj.764)
Supplement: Supplementary file 4 — Appendix S4. ANOVAs on contributed amount of money for the common project and on the estimated level of money Amount contributed by the co‐actor for the common project. [file PCHJ-13-749-s003.docx]

**Appendix D: ANOVAs on contributed amount of money for the common project and on the estimated level of money Amount contributed by the co-actor for the common project.**

**Table 3.** *ANOVA for social mindfulness, Zhongyong thinking style, gender, and student status on contributed amount of money for the common project.*

| Source | *df* | *MS* | *F* | *p* | η²_P_ |
| --- | --- | --- | --- | --- | --- |
| Social Mindfulness (SoMi) | 1 | 69.70 | 0.370 | .544 | .004 |
| Zhongyong thinking style (ZTS) | 1 | 1009.85 | 5.360 | .023 | .049 |
| Gender (G) | 1 | 209.26 | 1.111 | .294 | .011 |
| Student (S) | 1 | 356.95 | 1.895 | .172 | .018 |
| SoMi x ZTS | 1 | 60.11 | 0.319 | .573 | .003 |
| SoMi x G | 1 | 0.07 | 0.000 | .985 | .000 |
| SoMi x S | 1 | 18.73 | 0.099 | .753 | .001 |
| ZCTS x G | 1 | 839.93 | 4.458 | .037 | .041 |
| ZCTS x S | 1 | 493.83 | 2.621 | .108 | .025 |
| G x S | 1 | 374.93 | 1.990 | .161 | .019 |
| SoMi x ZTS x G | 1 | 225.92 | 1.199 | .276 | .011 |
| SoMi x CZTS x S | 1 | 29.59 | 0.157 | .693 | .002 |
| SoMi x G x S | 1 | 0.29 | 0.002 | .969 | .000 |
| CZCTS x G x S | 1 | 405.35 | 2.152 | .145 | .020 |
| SoMi x CZTS x G x S | 1 | 579.48 | 3.076 | .082 | .029 |
| Within-cells errors | 104 | 188.40 |  |  |  |

*Note.* All *p*'s are for two-tailed tests; *R^2^* = .120.

**Table 4.** *ANOVA for social mindfulness, Zhongyong thinking style, gender, and student status on the amount of money estimated to be contributed by the interaction partner for the common project.*

| Source | *df* | *MS* | *F* | *p* | η²_P_ |
| --- | --- | --- | --- | --- | --- |
| Social Mindfulness (SoMi) | 1 | 741.07 | 4.422 | .038 | .041 |
| Zhongyong thinking style (ZTS) | 1 | 4245.93 | 25.337 | **< .001** | .196 |
| Gender (G) | 1 | 794.71 | 4.742 | **.032** | .044 |
| Student (S) | 1 | 211.01 | 1.259 | .264 | .012 |
| SoMi x ZTS | 1 | 21.97 | 0.131 | .718 | .001 |
| SoMi x G | 1 | 29.33 | 0.175 | .677 | .002 |
| SoMi x S | 1 | 4.09 | 0.024 | .876 | .000 |
| ZTS x G | 1 | 1154.74 | 6.891 | **.010** | .062 |
| ZTS x S | 1 | 241.70 | 1.442 | .232 | .014 |
| G x S | 1 | 231.57 | 1.382 | .242 | .013 |
| SoMi x ZTS x G | 1 | 899.09 | 5.365 | .**023** | .049 |
| SoMi x ZTS x S | 1 | 1.38 | 0.008 | .928 | .000 |
| SoMi x G x S | 1 | 2.77 | 0.017 | .989 | .000 |
| ZTS x G x S | 1 | 155.09 | 0.925 | .338 | .009 |
| SoMi x ZTS x G x S | 1 | 28.24 | 0.169 | .682 | .002 |
| Within-cells errors | 104 | 167.58 |  |  |  |

*Note.* All *p*'s are for two-tailed tests; *R^2^* = .292.
